# Selective effects of exercise on reactive and proactive inhibition in Parkinson’s disease

**DOI:** 10.7717/peerj.13628

**Published:** 2022-06-23

**Authors:** Zhen Wang, Yan-Ling Pi, Yin Wu, Jianing Wei, Yuting Li, Jian Zhang, Zhen Wang

**Affiliations:** 1 School of Exercise and Health Science, Xi’an Physical Education University, Xi’an, China; 2 School of Psychology, Shanghai University of Sport, Shanghai, China; 3 Shanghai Punan Hospital of Pudong New District, Shanghai, China; 4 School of Economics and Management, Shanghai University of Sport, Shanghai, China; 5 School of Nursing, Anhui University of Chinese Medicine, Hefei, China; 6 School of Martial Arts, Shanghai University of Sport, Shanghai, China

**Keywords:** Parkinson’s disease, Motor inhibition, Physical exercise, Stop signal

## Abstract

**Objective:**

Patients with Parkinson’s disease (PD) have an obvious motor inhibition disorder, which is closely related to their motor symptoms. Although previous studies have shown that exercise can improve their inhibition deficits, the effect of exercise on different types of inhibition (proactive and reactive inhibition) has not been addressed.

**Methods:**

We used a behavioral paradigm combined with a series of questionnaires to explore the effect of long-term exercise on different types of motor inhibition in 59 patients with PD aged 55–75 years. According to the intensity and frequency of exercise, the participants were divided into regular-exercise and no-exercise groups. To obtain the average reference value for inhibition ability at the same age, we also recruited 30 healthy elderly people as controls.

**Results:**

The main defect in the motor inhibition of PD is reactive inhibition, while proactive inhibition has no obvious differences compared with healthy controls. Additionally, compared with the non-exercise group, PD in the exercise group showed significantly better reaction speeds and reactive control ability, fewer motor symptoms and negative emotions.

**Conclusions:**

Taken together, the motor inhibition defects of patients with PD affect only reactive inhibition. In addition, PD with exercise reported fewer negative emotions than that of the non-exercise group, indicating that exercise can relieve negative emotions and improve behavioral symptoms and quality of life in PD to a certain extent. We demonstrate for the first time that exercise has and can improve reactive inhibition in PD patients and has no effect on proactive inhibition.

## Introduction

Motor inhibition, a central component of inhibitory control (Wöstmann et al., 2013), is the ability to effortfully withhold or cancel a routine, initiated or otherwise, through pre-potent motor responses (Hampshire, 2015; Cowie, 2017). It can be classified as reactive and proactive inhibition, which fall into distinct neuropsychological domains (Aron, 2011). Reactive inhibition is the canceling of an ongoing reaction immediately upon the presentation of a stop signal, whereas proactive inhibition involves preparation for stopping an action when necessary (*i.e.*, adjustment of the action strategy in advance, according to the environment in which the stop signal will appear) (Aron, 2011; Mirabella, 2021). Although Parkinson’s disease (PD) causes obvious motor inhibition defects (Gauggel, Rieger & Feghoff, 2004; Bokura, Yamaguchi & Kobayashi, 2005; Antonelli, Ray & Strafella, 2010; Wylie et al., 2010; Obeso et al., 2011; Bloemendaal et al., 2018; Pan et al., 2018), no consensus has been reached regarding the specific manifestations of these defects in patients with the disease. Some researchers believe that PD is associated with poor proactive and reactive inhibition (Obeso et al., 2013; Jana et al., 2020), whereas others found that this defect appears only in reactive inhibition control (Di Caprio et al., 2020). Therefore, one of the purposes of this study is to determine what motor inhibition defects in PD actually exist.

Mainstream dopamine drugs do not successfully alleviate the motor symptoms of response inhibition deficit in PD (Schubert et al., 2002; PD MED CollaborativeGroup, 2014). Long-term exercise can delay the decline of executive function, enabling patients to obtain additional cognitive benefits in terms of inhibitory control (Voelcker-Rehage, Godde & Staudinger, 2011; Drollette et al., 2014). Exercise therapy can also delay the deterioration of motor function (Goodwin et al., 2008; Dibble, Addison & Papa, 2009; Duchesne et al., 2015) and is a feasible method for the effective improvement of inhibitory control in the clinical setting (Chang et al., 2012; Drollette et al., 2014). However, the effects of exercise on different types of motor inhibition defect in patients with PD have not been examined. Hence, another major objective of this study was to explore the influence of exercise on different motor inhibition defects in patients with PD, and to provide theoretical support for precise exercise prescriptions in the future.

At present, there are mainly go/no-go (GNG) (Donders, 1969) and stop-signal tasks (SST) (Logan, Cowan & Davis, 1984) behavioral paradigms in motor inhibition research. The GNG task measures an individual’s ability to suppress a potential action (action restraint), and the SST measures their ability to inhibit an action that has been initiated (action cancellation) (Mirabella, 2021). Reactive inhibition is quantified by measuring the stop-signal reaction time (SSRT), or the time it takes to inhibit an action when a stop signal is presented. Proactive inhibition is measured by determining the response delay effect (RDE). In this study, we used a modified SST (Ye et al., 2014) to compare reactive and proactive inhibitory control in patients with PD and age-matched healthy controls, to identify the subdomains in which the motor inhibitory defects of PD are reflected.

This study was conducted with healthy elderly adults, and patients with PD who did and did not exercise regularly. A response inhibition task consisting of randomly ordered trials (75% go, 8% no-go, and 17% stop signal trials) was administered to the study participants to explore the effects of exercise on different types of motor inhibition caused by PD. We hypothesized that exercise would help to improve patients’ reactive and proactive motor inhibition.

## Methods

### Participants

Subjects were recruited for this study from the outpatient neurology department of Shanghai Punan Hospital, Pudong New District, Shanghai, China. Before recruiting, we used G*Power software to count each group. The results indicated that 66 participants (22/group) will be required to demonstrate exercise effects on behavioral outcomes with variance (ANOVAs) at a 0.05 significance level and 80% power. We recruited 59 patients aged 55–75 years with idiopathic PD and no family history of Parkinsonism; these patients were evaluated using the Unified Parkinson’s Disease Rating Scale part III (UPDRS-III) and had the stages at I–III of Hoehn and Yahr. According to the Edinburgh Handedness Inventory, all participants were right-handed and had normal or corrected-to-normal vision. The Montreal Cognitive Assessment (MoCA) was administered to assess participants’ overall cognitive function. Participants with neurological or psychiatric diseases other than PD, those with symptoms of impulse control disorder, and those who were unable to stand and walk without an assistive device were excluded. The patients’ course of disease, age, behavioral symptoms, and other variables were well matched and controlled. The International Physical Activity Questionnaire (IPAQ) was used to collect data on subjects’ weekly physical activity. Patients with PD who exercised more than three times per week (>60 min/episode) were assigned to the regular exercise group, and others were assigned to the no-regular-exercise group. Participants followed their regular medication regimens during the study period. We collected data on patients’ medication use, including dosages, for control of the statistical analyses. To obtain a standard measure of motor inhibition and to enable the examination of differences in reactive and proactive inhibition between healthy elderly individuals and people with PD, we also recruited 30 age matched healthy right-handed elderly participants. All procedures were performed according to the Declaration of Helsinki and approved by the Shanghai University of Sport Ethics Committee (102772020RT107). All candidates were required to provide written informed consent prior to study inclusion.

### Procedure

The procedure consisted of the collection of demographic information and administration of questionnaires and response inhibition tasks, which took about 1.5 h per participant. The questionnaires and behavioral tasks used in this study are the same as those used by Wang et al. (2021), which are part of the methodology in our published protocol.

The Parkinson Disease Sleep Scale (PDSS), Hospital Anxiety and Depression Scale (HADS), 39-item Parkinson Disease Questionnaire (PDQ-39), and Timed Up-and-Go Test (TUGT) were administered in counterbalanced order only in PD. The inhibition tasks performed by the PD and healthy control groups, including the maybe and never tasks, were randomly cross-checked within and between groups.

**Figure 1:**
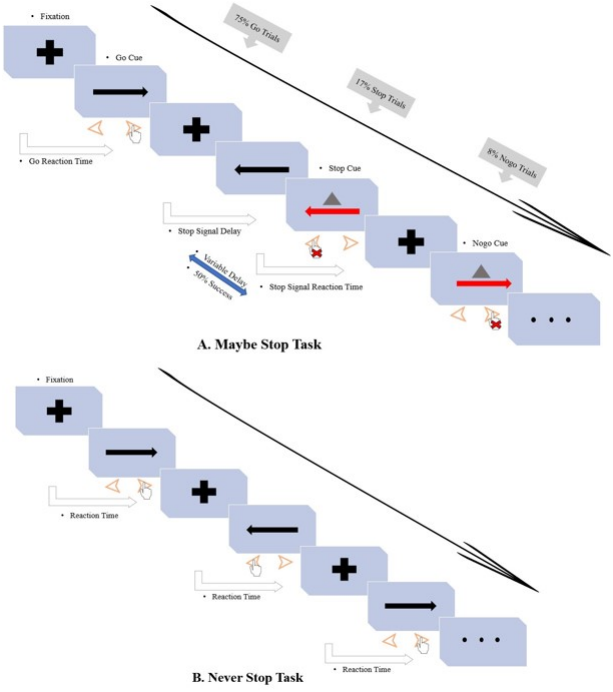
Diagram of the motor response inhibition task. Subjects sat in a relaxed position (with the elbows, hips, and knees flexed at 90–100°) in front of a computer with a monitor in their line of sight at a distance of 75 cm. Before the test, the subjects were instructed about the experiment and completed a practice test to become familiar with the experimental task. Then they entered the formal experiment stage and randomly completed two experimental tasks. (A) Maybe stop task (pseudorandom mix of 75% go trials, 17% stop trials, and 8% no-go trials). The gray triangle served as the visual stop signal. (B) Never stop task (360 go-only trials).

The maybe stop task (MST) was administered as a pseudorandom combination of 75% go trials, 17% stop trials, and 8% no-go trials (total: 480 trials in four blocks; Fig. 1A). This task enabled us to examine both types of inhibition while matching for medication dosages, practice effects, and fatigue. In go trials, subjects responded to left- and right-pointing black arrows (displayed for 1,000 ms) by pressing corresponding buttons with the right index finger. In stop trials, responses were cued initially by left- and right-pointing black arrows, followed by a red arrow with a gray triangle (requiring nonresponse) after a stop-signal delay (SSD). The SSD (initial duration, 250 ms) was varied from trial to trial to adjust the task difficulty using a stepwise algorithm; it was increased by 50 ms following successful nonresponse and decreased by 50 ms following failed nonresponse (Benis et al., 2014) to maintain 50% successful inhibition. To prevent participants from deliberately slowing down their reaction time to increase the chance of correct stopping (a common strategy used to increase the probability of successful stop), we were told before the task that the stopping success rate was about 50% regardless of whether the response time was deliberately delayed. SSRT based on independent horse racing model assumptions is estimated using an integral method, which estimates SSRT by subtracting the average SSD from the mean RT (Logan, Cowan & Davis, 1984). In no-go trials, subjects were required to make no response to a red arrow with a gray triangle (displayed for 1,000 ms), in a setup equivalent to a 0-ms SSD. In no-go trials, action selections and restraint mechanisms are needed to prevent a prepotent response, whereas in stop trials, the initial response is cancelled and inhibition of motor action is induced.

The never stop task (NST), a reaction time task including only go trials, was administered in three blocks (total: 360 trials; Fig. 1B) to measure participants’ general alertness and motor speed when pressing buttons with the knowledge that no stop signal would be presented. We asked the participants to respond as quickly as possible to the stimuli presented.

The SSRTs reflect the reactive inhibition (Kleerekooper et al., 2016), while the proactive inhibition was assessed by comparing the RTs from no-stop MST trials and go-only NST trials to assess the context effect. Therefore, we choose the combination of two behavioral tasks to detect among three groups.

### Statistical analysis

All data were analyzed using SPSS software (version 25.0). Go response times (RTs), SSRTs, go errors (pressing of the wrong button or missing the button), and no-go errors (pressing of a button) were taken as behavioral parameters (Ye et al., 2014). Trials with RTs shorter or longer than the mean ±3 standard deviations (SDs) were considered to be outliers and were excluded from the analysis.

We assessed the normality of data distribution before processing using the Shapiro–Wilk test; all data were distributed normally. For continuous variables (*e.g.*, PDSS, PDQ-39, TUGT, and HADS scores), the independent-sample *t* test was used to evaluate differences between PD groups. Considering that drugs and disease severity may affect behavioral results of PD, LEDD, UPDRS-III and disease duration were first taken as covariables to conduct analysis of covariance (ANCOVA) on behavioral data of PD between two groups. Then, analysis of variance (ANOVA) was applied to assess the SSRTs and RTs changes in context effect among the three groups. Bonferroni correction was performed for all multiple comparisons. To further verify the effect of exercise on reactive inhibition, Pearson and Spearman analyses were performed to assess the correlation of SSRTs with the physical activity level and UPDRS-III score. Data are expressed as means ± SDs.

## Results

### Participant characteristics

Out of the 89 recruited participants, three in the PD non-exercise group and one in the PD exercise group were excluded because they did not meet the performance standard of 80% accuracy of correct response in the NST. Three patients in the PD no-exercise group who failed to complete all tests for health reasons were also excluded. Totally, data from 82 participants (28 in the PD exercise group, 24 in the PD no-exercise group, and 30 healthy controls) were included in the final analysis. The participants’ demographic and clinical characteristics are summarized in Table 1. Among these characteristics, only the UPDRS-III score was differed significantly, between the PD groups.

**Table 1:** Demographic and clinical features of the participants.

	Exercise group (28)	Non-exercise group (24)	Control group (30)
Age	68.21 ± 5.07	68.46 ± 4.40	66 ± 4.89
Male: Female	9:19	10:14	13:17
Educational level	13 ± 2.62	11.95 ± 2.54	12.43 ± 2.33
MoCA	27.18 ± 1.52	26.25 ± 2.13	27.03 ± 1.47
UPDRS-III	16 ± 6.88	25 ± 12.58	/
Levodopa (mg)	270.09 ± 149.32	359.38 ± 187.27	/
Duration of disease	6.45 ± 3.65	5.58 ± 3.87	/
Hoehn & Yahr stage	1.16 ± 0.36	1.29 ± 0.44	/

**Notes.**

MoCA Montreal Cognitive Assessment UPDRS-III Unified Parkinson’s Disease Rating Scale part III

### Reactive inhibition

**Figure 2:**
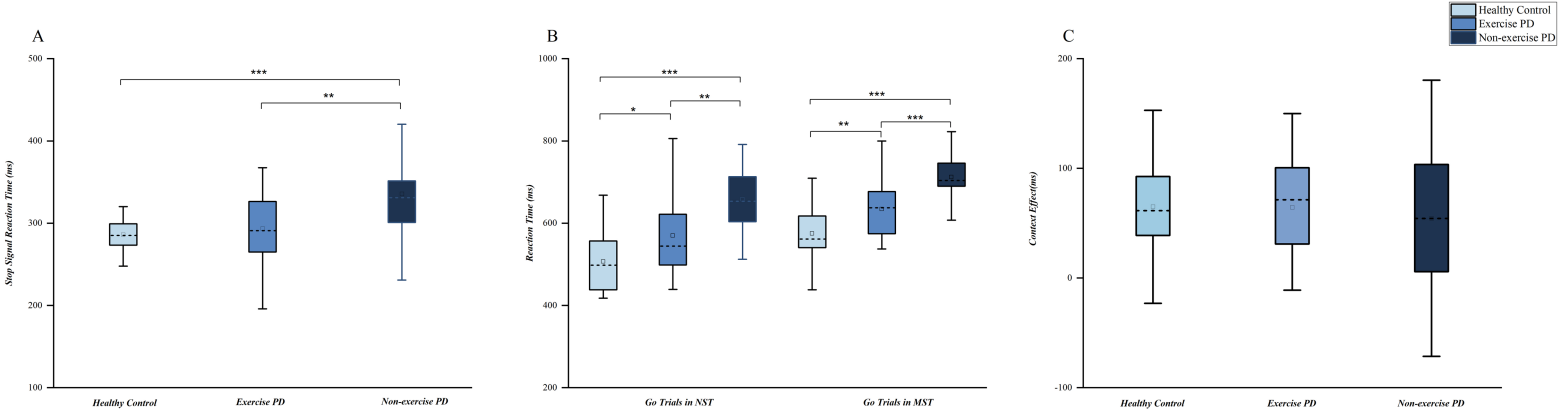
Differences in reactive and proactive motor inhibition between groups. (A) Reactive inhibition. Box plot of stop-signal reaction times. (B) Box plot of response times for no-stop and go-only trials in the MST and NST. (C) Proactive inhibition. Average context effect values. PD, Parkinson’s disease; NST, never stop task; MST, maybe stop task. ^∗^*p* < 0.05, ^∗∗^*p* < 0.01, ^∗∗∗^*p* < 0.001.

First of all, we reviewed the data to explore whether the stepwise algorithm for SSD adjustment is applicable to all study groups, and one-way ANOVA about stop ACC showed no difference among the three groups (*F* (2.79) = 2.689, *p* = 0.074, partial *η*^2^ = 0.064). First, the results of ANCOVA showed that LEDD (*p* = 0.308), UPDRS-III (*p* = 0.309) and duration of disease (*p* = 0.873) had no bias on the SSRT of PD. Then, ANOVA revealed significant differences in SSRTs among the three groups (*F* (2,79) = 9.741, *p* <  0.001, partial *η*^2^ = .198). Bonferroni *post-hoc* analysis showed that they were significantly longer in the PD no-exercise group than in the healthy control group (*p* < 0.001) and the PD exercise group (*p* = 0.002). SSRTs did not differ significantly between the PD exercise and healthy control groups, although they tended to be shorter in the latter (Table 2, Fig. 2A). To quantify our SSRT results between the healthy control group and PD exercise group, we conducted a Bayesian *t*-test with JASP, the results showed that there is consistent evidence in support of the null hypothesis (BF_10_ = 0.371). This result further confirms that exercise can not only effectively improve the reactive inhibition of patients with PD, but that long-term adherence to an exercise regimen can improve reactive inhibition to the same level as seen in healthy elderly people.

**Table 2:** SSRT, RT, and ACC results.

	Exercise group (28)	Non-exercise group (24)	Control group (30)	*p*	*η* ^2^_*p*_
Go RTs (NST)	569.97 ± 101.5^**^	657.65 ± 69.85^†††^	506.73 ± 73.41^#^	<0.001	.375
Go RTs (MST)	634.43 ± 71.27^***^	712.01 ± 61.63^†††^	574.89 ± 67.43^##^	<0.001	.413
SSRT	293.51 ± 41.64^**^	335.64 ± 63.30^†††^	286.06 ± 18.39	<0.001	.198
Stop Acc	0.52 ± 0.02	0.52 ± 0.01	0.51 ± 0.02	.074	.064
ACC (NST)	0.95 ± 0.02	0.95 ± 0.03^†††^	0.98 ± 0.02^##^	<0.001	.203
ACC (MST)	0.95 ± 0.04	0.93 ± 0.04^†††^	0.98 ± 0.0^##^	<0.001	.309
NoGo Error	0.21 ± 0.69	0.29 ± 0.75	0.53 ± 0.94	.295	.030

**Notes.**

ACC was defined as the ratio between the number of correct responses and the total number of trials presented, calculated from the sum of correct responses. Trials in which the participants missed the target, pressed the button incorrectly, or waited longer than the stimulus presentation in order to improve their stop ACC were excluded as errors.

SSRT stop-signal reaction time RT reaction time ACC accuracy NST never stop task MST maybe stop task Stop Acc probability of a successful stop in the MST

*Post-hoc* analysis results: no-exercise PD vs. healthy control group. ^††^*p* < 0.01, ^†††^*p* < 0.001; exercise PD vs. healthy control group: ^#^ *p* < 0.05, ^##^ *p* < 0.01, ^###^ *p* < 0.001; exercise PD vs. no-exercise PD group: ^∗^*p* < 0.005, ^∗∗^*p* < 0.01, ^∗∗∗^ *p* < 0.001.

### Proactive inhibition

Firstly, we analyzed the RT of go trials in MST and NST, and the results showed that the reaction time of the three groups was significantly different, namely, the reaction time of HC group was the fastest, while PD without exercise group was the slowest (Fig. 2B). Proactive inhibition was assessed by comparing the RTs from no-stop MST trials and go-only NST trials to assess the context effect, which described as motor restraint in response to contextual cues indicating increased stop-signal probability. The results of ANCOVA showed that LEDD (*p* = 0.137), UPDRS-III (*p* = 0.730) and duration of disease (*p* = 0.804) had no bias on the context effect of PD. The context effect did not differ among groups (Fig. 2C). Additionally, no difference was found in no-go ACC among groups in this study (*p* = 0.295).

### Questionnaire results

Questionnaire scores are provided in Table 3. The HADS and PDQ-39 scores were significantly lower in the PD exercise group than in the PD no-exercise group (*t* (50) = −2.381, *p* = 0.021 and *t* (50) = −3.086, *p* = 0.003, respectively). TUGT results also differed significantly between groups (*t* (50) = −5.339, *p* < 0.001); PDSS scores did not (*t* (50) = 0.249, *p* = 0.804). These results indicate that long-term exercise can improve the comprehensive ability of PD patients. Although it has no obvious effect on sleep quality, it has a huge impact on mood which can significantly reduce the generation of negative emotions.

**Table 3:** Questionnaire scores.

	Exercise group (28)	Non-exercise group (24)	t	*P*
HADS	6.43 ± 5.19	10.42 ± 6.87	−2.381	.021
PDSS	115.43 ± 16.06	114.21 ± 19.27	.249	.804
PDQ-39	22.75 ± 11.82	34.54 ± 15.68	−3.086	.003
TUGT	9.08 ± 1.41	12.11 ± 2.59	−5.339	<.001

**Notes.**

HADS Hospital Anxiety and Depression Scale PDSS Parkinson Disease Sleep Scale PDQ-39 39-item Parkinson Disease Questionnaire TUGT Timed Up-and-Go Test

### Correlation of SSRTs with the physical activity level and UPDRS-III

**Figure 3:**
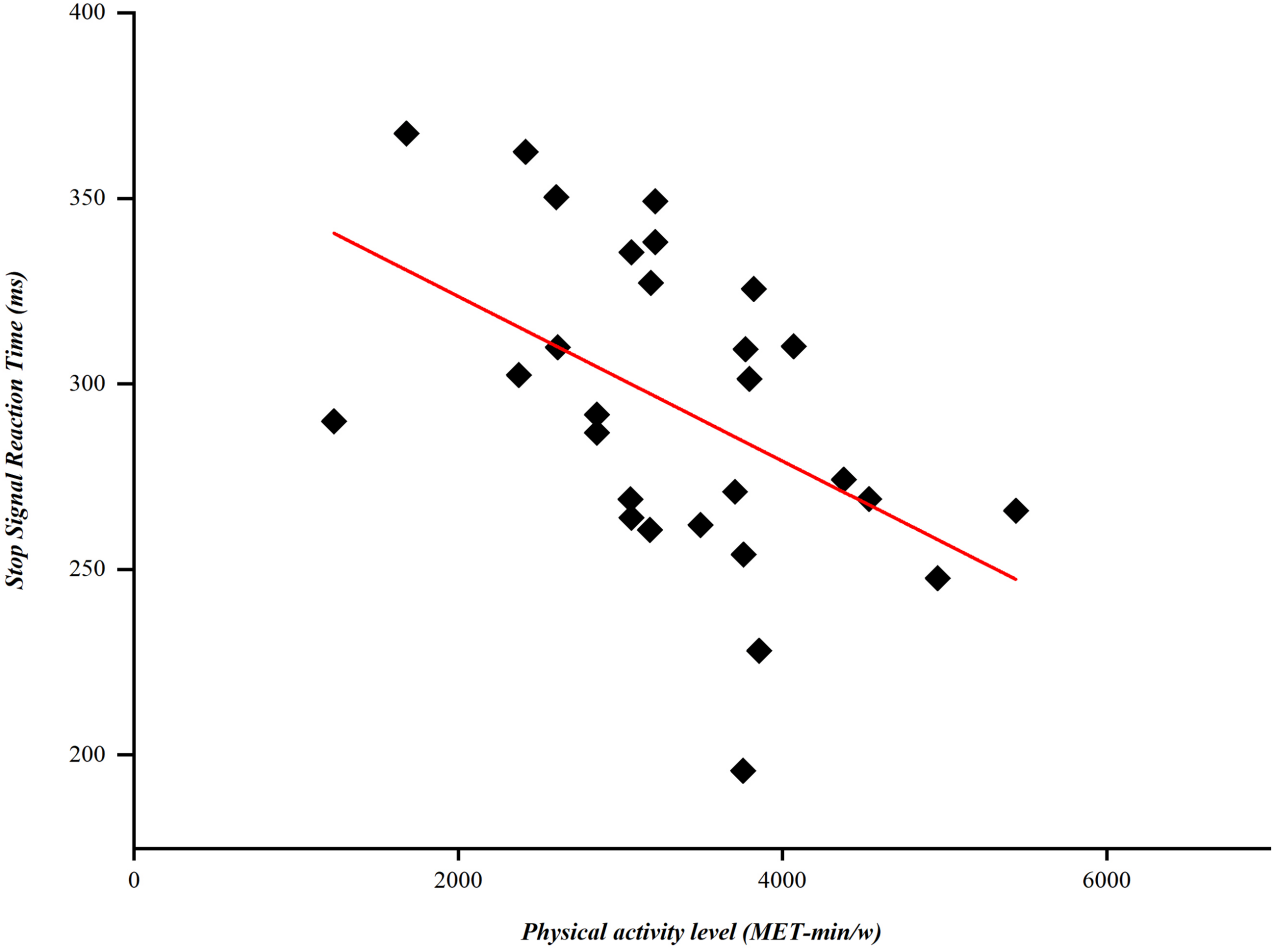
Correlation between stop-signal reaction times (SSRTs) and physical activity levels.

Due to the significant differences in reactive inhibition between PD groups, we analyzed the correlation between physical activity and motor symptoms using the SSRTs from the PD exercise group. Pearson correlation analysis revealed a negative correlation between physical activity levels and SSRTs (*r* = −0.484, *p* = 0.009), with higher physical activity levels correlated with shorter SSRTs (Fig. 3). Further to assess the effects of motor symptoms on reaction speed and inhibitory control in patients with PD, we performed Spearman analysis of correlations between the UPDRS-III score and SSRTs and RTs. Spearman analysis revealed that patients with PD with shorter SSRTs had lower UPDRS-III scores (*r* = 0.278, *p* = 0.046); no correlation with RTs was observed.

## Discussion

In this study, the modified stop-signal behavioral task and clinical questionnaires were used to investigate that the motor inhibition deficiency occurring in PD was mainly reactive; no significant difference in proactive inhibition compared with healthy elderly adults was observed. Additionally, long-term exercise obviously improved the reactive inhibition of patients with PD.

### PD inhibition defects are reflected mainly in reactive inhibition control, with no significant change in proactive inhibition

Through SSRT analysis, we found that patients with PD could not immediately reactivate and apply the currently needed cue information when using cognitive control to resolve conflicts, meaning that they showed significant reactive inhibition deficiency, consistent with previously reported results (Obeso et al., 2013; Di Caprio et al., 2020). Such damage, reflected in behavior, is due to changes in brain activity patterns in the executive control region, including the prefrontal cortex (PFC), which is one of the most important brain regions of the inhibition control system (MacDonald et al., 2000; Miller & Cohen, 2001). The brain neurotransmission system and frontal-basal ganglia (BG) network constitute the main physiological basis for the regulation of the interaction between cognition and motor inhibition in elderly adults (Levin & Netz, 2015); as a result of physical PD symptoms associated with PFC damage, from a brain structural perspective the reactive inhibition deficiency in patients with PD is a physiological handicap. Furthermore, the BG cortical pathway is the anatomical and physiological basis of daily motor behavior regulation, and it interacts with PFC regions for inhibitory control. The motor cortical-hypothalamic nucleus–globus pallidus neural pathways inhibit motor processes globally as hyper-direct pathways in the BG (Aron et al., 2007; Jian & Shenquan, 2020), its conduction is related to the subthalamic nucleus (STN). The STN constitutes the BG dopaminergic system and plays a role in signal intensification. Due to pathological damage of the STN and the imbalance among different dopamine receptors, patients with PD have difficulty receiving information input from brain regions and sending stop signals to intercept BG output, which manifests as inhibitory control defects. In addition, these patients have dopamine deficiency, the main cause of reactive inhibition deficiency. Throughout the neural signal transmission process, midbrain dopamine system gating signals entering PFC representation during inhibitory control, and only related cues are allowed to continue to participate (Braver & Cohen, 2000; O’Reilly et al., 2002). The dopamine phase burst is an important prerequisite for the maintenance of PFC representation information. Due to the degeneration of neurons with the lack of dopamine production, patients with PD show only by a high dopamine phase burst and early PFC activation in the early stage of signal transmission, meaning that their proactive inhibition is similar to that of healthy controls. In the reactive inhibition phase with no dopamine phase burst, PFC activation fades quickly and detection cues cannot continue to be represented (Xu, Tang & Chen, 2013), resulting in marked weakening of the reactive inhibition ability.

As top-down cue-driven information processing, proactive inhibition is the ability to select attentional process task-related cues and to actively represent and maintain them at a subsequent time (Braver et al., 2009). From the perspective of the dual-task requirement, the reason for the lack of a difference between groups in the proactive response in this study may also be the increase in cognitive demand. In contrast to the response to the go signal in the NST, that in the MST had to be performed while subjects monitored the stop signal; this increased working memory load could disperse subjects’ visual attention, leading to increased RTs in the MST among healthy people and those with PD (Verbruggen & Logan, 2009). Proactive inhibition deficits are more common in individuals with emotion-related psychiatric disorders (Aron, 2011; Obeso et al., 2013), such as schizophrenia (Zandbelt et al., 2011) and bipolar disorder (Robinson et al., 2013). Studies have found that negative emotions in PD mostly occur in the advanced stage of the disease (Chaudhuri & Schapira, 2009); all the participants in this study had mild to moderate PD, so we did not observe emotional problems. The inconsistency in results may also be attributed to physiological differences among groups. Subjects with autism spectrum disorder (ASD) (Mancini et al., 2018) have only proactive defects, with no difference in reactivity, supporting the above conjecture. In addition, our subjects were elderly adults. Cognitive inhibition becomes problematic with aging, manifesting mainly in the level of defect in proactive inhibition (Braver & Barch, 2002; Friedman, 2014; Hartmann et al., 2019), which has the same U-shaped developmental trend as individual physiological changes (*i.e.*, increasing initially stage and gradually declining with age after peaking) (Xu, Tang & Chen, 2013). Interestingly, Burgess found that proactive inhibition is associated with fluid intelligence, and that people with high fluid intelligence have stronger proactive inhibition. This finding also provides evidence that proactive inhibition is related to development and aging, as fluid intelligence also decreases with age (Burgess et al., 2011).

### Long-term exercise at a certain intensity can improve the response inhibition ability in patients with PD

Long-term repetitive training results in sustained functional and morphological changes in the brain ((Eleanor, Katherine & Hugo, 2006); Rabipour & Raz, 2012), indicating that the adult brain is neuroplastic, even in advanced age (Liu et al., 2019). Braver showed that the cognitive control system, including the ability to predict cues, of elderly adults under training gradually becomes consistent with that of ordinary adults (Braver et al., 2009). As an important part of executive control, motor inhibition gives full play to its plasticity (Xu, Tang & Chen, 2013), which is significantly improved through training and accompanied by PFC activation (Davis et al., 2011; Brevers et al., 2018). For example, high-level athletes have better cognitive and motor resource mobilization abilities when facing new situations, and have significant advantages in inhibitory control and decision-making processes (Brevers et al., 2018). The activation of the lateral PFC (Wykes et al., 2002) can be altered by training in schizophrenic individuals with inhibition deficiency to improve their proactive inhibition ability (Edwards, Barch & Braver, 2010).

Albares showed that PD motor symptoms are related closely to decreased inhibitory ability (Albares et al., 2015), and exercise as a means of regulation has been shown to effectively improve executive control ability in patients with PD (Smith et al., 2010; Drollette et al., 2014; Caciula et al., 2016; Fiorelli et al., 2019). Most clinical motor manifestations of PD, such as bradykinesia and rigidity, can be alleviated effectively by exercise (Yang et al., 2014; Duchesne et al., 2015; Hampshire, 2015; Schenkman et al., 2018; Silva-Batista et al., 2018; Kwok et al., 2019; Leal et al., 2019); these findings are supported by the significantly lower UPDRS-III scores in PD exercise group than in the PD no-exercise group in this study. In addition, the physical activity level correlated negatively with SSRTs, indicating that the performance of a certain amount of weekly physical activity for a long time had significantly improved the reactive inhibition ability of patients with PD. Exercise improves dopamine neurotransmission and promotes dopamine release by regulating dopamine receptors (Fisher et al., 2008; Beeler et al., 2010), which appears to compensate to a certain extent for the loss of dopamine neurons in patients with PD due to substantia nigra density (Lees, Hardy & Revesz, 2009; Mazzoni, Shabbott & Cortés, 2012) and pathological defects with dopamine dependence disorder as the main factor (Ferrazzoli et al., 2018). Moreover, dopamine has been shown to be involved in the regulation of response inhibition (Ghahremani et al., 2012); thus, exercise can improve reactive inhibition in patients with PD.

Our results show that long-term exercise can improve only reactive inhibition deficiency, and only to some extent. Specific training has been found to improve proactive inhibition defects in patients with schizophrenia, with no effect on reactive inhibition (Edwards, Barch & Braver, 2010), but studies on athletes show that exercise training not only promotes reactive inhibition, but also promotes proactive inhibition. We speculate that biological differences among participant groups are the main reasons for these inconsistencies, as the major deficit in schizophrenia affects proactive, rather than reactive, inhibition, and the opposite is true for PD. In addition, we observed significant differences in PDQ-39 and HADS scores, related to emotion, between PD groups in this study. Relative to the PD no-exercise group, the PD exercise group had a significantly higher level of positive emotion and significantly better expression of exercise ability and symptoms. Exercise is an auxiliary therapy that benefits physical and mental development, enhances social communication ability, and promotes the generation of positive emotions. Furthermore, positive emotions temporarily improve dopamine release, enhance the flexibility of working memory representation, and improve reactive control, with no true effect on proactive control (Van Wouwe, Band & Ridderinkhof, 2011).

By exploring the efficacy of exercise therapy on PD inhibition, we found that on the one hand, patients’ self-esteem and self-confidence can be significantly improved, and through participation in intensive social sports activities, they can further regain control of their body and behavior with confidence, reduce possible behavioral impulses, and improve the quality of life. On the other hand, when faced with emergencies, they can more effectively control their own behaviors, reduce risks and ensure safety. Since inhibition is closely related to motor symptoms, exercise can further reduce motor symptoms, relieve negative emotional states, and improve life happiness. Therefore, we believe that the intervention of exercise on PD inhibition defect can better facilitate the rehabilitative benefits of PD physically and mentally.

### Limitations

Although this is the first study to examine the effect of long-term exercise on the improvement of inhibition deficits in Parkinson’s disease from different dimensions of motor inhibition, there are some limitations that need to be taken into account when interpreting the results. First of all, we defined the patient groups only on the basis of IPAQ and patients’ subjective self-reported data, without a fixed exercise training method for intervention evaluation. Although we recruited subjects to exercise at a frequency of 60 min or more three times a week for more than a year, these reports were from the patients themselves, which did not enable us to distinguish their exercise intensity more accurately. Moreover, we didn’t make a specific distinction between the exercise types of PD recruited in the exercise group, and there was a mixture of various exercise styles. Individual and group exercise may have different effects. The research from Docu Axelerad et al. (2022) showed that group interactive movement (tango) significantly promoted the improvement of PD motor and non-motor symptoms in the changeable scenes of social environment and interpersonal communication. Therefore, future longitudinal studies should be more specific on exercise intervention types effect. Secondly, the size and representativeness of our sample volume are not strong enough. Although we strictly followed the regional randomness in the selection of subjects, there may still be some defects that are not enough to match with large samples. In future studies, we will continue to explore this issue from a neuro-electrophysiological perspective through longitudinal intervention follow-up.

## Conclusions

This study showed that the motor inhibition control disorder in patients with PD affects only reactive inhibition. In addition, long-term exercise can not only significantly improve these patients’ reactive inhibition ability, but also improve their behavioral motor performance and reduce the occurrence of negative emotions to a certain extent. Our results provide a theoretical basis for the formulation of long-term rehabilitation exercise prescription for PD.

## Supplemental Information

10.7717/peerj.13628/supp-1 Supplemental Information 1 Raw data

## Additional Information and Declarations

### Data Availability

The following information was supplied regarding data availability: The raw data are available in the Supplementary Files.
